# Ectomesenchymal *Six1* controls mandibular skeleton formation

**DOI:** 10.3389/fgene.2023.1082911

**Published:** 2023-02-08

**Authors:** Songyuan Luo, Zhixu Liu, Qian Bian, Xudong Wang

**Affiliations:** ^1^ Department of Oral and Craniomaxillofacial Surgery, Shanghai Ninth People’s Hospital, Shanghai Jiao Tong University School of Medicine, Shanghai, China; ^2^ National Center for Stomatology, National Clinical Research Center for Oral Diseases, Shanghai Key Laboratory of Stomatology, Shanghai, China; ^3^ Shanghai Institute of Precision Medicine, Shanghai, China

**Keywords:** Six1, craniofacial development, mandibular skeletal development, cranial neural crest cells, osteogenic differentiation

## Abstract

Craniofacial development requires intricate cooperation between multiple transcription factors and signaling pathways. Six1 is a critical transcription factor regulating craniofacial development. However, the exact function of Six1 during craniofacial development remains elusive. In this study, we investigated the role of Six1 in mandible development using a *Six1* knockout mouse model (*Six1* ^*−/−*^) and a cranial neural crest-specific, *Six1* conditional knockout mouse model (*Six1* ^*f/f*^ *; Wnt1-Cre*). The *Six1* ^*−/−*^ mice exhibited multiple craniofacial deformities, including severe microsomia, high-arched palate, and uvula deformity. Notably, the *Six1* ^*f/f*^ *; Wnt1-Cre* mice recapitulate the microsomia phenotype of *Six1* ^*−/−*^ mice, thus demonstrating that the expression of *Six1* in ectomesenchyme is critical for mandible development. We further showed that the knockout of *Six1* led to abnormal expression of osteogenic genes within the mandible. Moreover, the knockdown of *Six1* in C3H10 T1/2 cells reduced their osteogenic capacity *in vitro*. Using RNA-seq, we showed that both the loss of Six1 in the E18.5 mandible and Six1 knockdown in C3H10 T1/2 led to the dysregulation of genes involved in embryonic skeletal development. In particular, we showed that Six1 binds to the promoter of *Bmp4*, *Fat4*, *Fgf18,* and *Fgfr2*, and promotes their transcription. Collectively, our results suggest that Six1 plays a critical role in regulating mandibular skeleton formation during mouse embryogenesis.

## Introduction

The craniofacial development of vertebrates is precisely regulated by various genes and signaling pathways, including BMP, FGF, and WNT (Yin et al., 2015; Graf et al., 2016). Most craniofacial tissues are derived from cranial neural crest cells (CNCCs), which arise from the dorsal central nervous system and migrate into the developing craniofacial region (Liao et al., 2022). Within maxillary and mandibular prominences, CNCCs differentiate into ectomesenchymal cells, and the ectomesenchymal cells subsequently differentiate into various cell and tissue types, including the frontonasal skeleton, bone and cartilage of the jaw and middle ear (Liao et al., 2022). In contrast to other mesoderm-derived bones of the skeleton, the mandibular skeleton is generated during mandibular development *via* an intramembranous process in which ectodermal mesenchymal cells aggregate and then differentiate into bone (Parada and Chai, 2015; Liao et al., 2022).

The intricate regulation of craniofacial development and differentiation requires a number of transcription factors, such as the MSX family, DLX family, and the SIX family transcription factors, among others (Alappat et al., 2003; Takechi et al., 2013). The SIX family is a group of evolutionarily conserved transcription factors, which are expressed in multiple organs of humans, mice, *drosophila*, and other organisms, and play an essential role in the development of the craniofacial skeleton, kidney, ear, nose, brain, muscle, and gonads (Serikaku and O'Tousa, 1994). The mammalian SIX family consists of six members (SIX1-6). SIX family genetic mutations lead to various deformities, including craniofacial deformities, hearing disorders, visual disturbance, renal hypoplasia, and muscular dysplasia. (Kumar, 2009).

Six1 has been demonstrated to be a crucial member of the SIX family transcription factors in the embryonic development (Wu et al., 2014; Liu et al., 2019). *Six1* knockout mice exhibited craniofacial deformity, hypoplastic kidneys (Xu et al., 2003), and severely dysplastic lungs (El-Hashash et al., 2011). Previous studies have shown that *Six1* exerts versatile transcription regulatory effects by interacting with different molecular partners. SIX1 can form a complex with EYA1 and activate transcription (Li et al., 2003). Moreover, SIX1 can also form a transcription complex with members of the DACH family and repress the expression of downstream genes (Li et al., 2003). Six1 regulates *Fgf10* and *Bmp4* expression in the otic vesicle and interacts with *Runx1* to regulate the cell fate of the Müllerian duct epithelium (Zheng et al., 2003; Terakawa et al., 2020).

It has been suggested that Six1 participates in the development of the craniofacial skeleton (Tavares et al., 2017). *Six1* is widely expressed in craniofacial tissues of different origins, such as ectoderm, mesoderm, and endoderm (Liu et al., 2019). *SIX1* mutation causes human branchio-oto-renal syndrome (BOR), characterized by hearing loss, auricular deformities, residual branchial arches, and renal abnormalities (Kumar et al., 2000; Ruf et al., 2004; Feng et al., 2021). However, the mechanisms by which SIX1 regulates craniofacial development, and skeletogenesis remain unclear.

In this study, we generated a *Six1* knockout mouse model and conditional deletion of *Six1* in cranial neural crest cells to investigate the role of Six1 in ectomesenchymal cells during murine embryonic mandibular development. We found that the mandibles of both *Six1* ^*−/−*^ and *Six1* ^*f/f*^ *; Wnt1-Cre* were significantly shortened, indicating that ectomesenchymal *Six1* participates in mandibular skeletal development. Combining RNA-seq and immunofluorescence staining, we demonstrated that mandibular osteogenesis is impaired in E18.5 and E16.5 *Six1* ^*−/−*^ mice. In particular, mRNA expression levels of several key osteogenesis-related genes, such as *Osteopontin* (*Opn*), *Osteocalcin* (*Ocn*) and *Osterix* (*Osx*), were found to be downregulated. *In vitro*, the knockdown of *Six1* in the mouse embryonic mesenchymal stem cell line C3H10 T1/2 resulted in decreased osteogenic differentiation capacity and dysregulation of ossification-related genes. By performing CUT&Tag, we further demonstrated that Six1 directly binds to the promoters of *Bmp4*, *Fgfr2*, *Fgf18,* and *Fat4*, all of which are critical genes involved in skeletal formation and regulates their expression (Hung et al., 2016; Crespo-Enriquez et al., 2019; Motch Perrine et al., 2019; Xu et al., 2021)*.* Taken together, our data suggest that Six1 plays a critical role in the regulation of ossification during embryonic mandibular skeletal development and elucidates the potential Six1-dependent gene regulation networks involved in mandibular development.

## Materials and methods

### Animals

The *Six1* knockout homozygous (*Six1* ^*−/−*^) and *Six1* conditional knockout (*Six1* ^*f/f*^) mouse models were generated using the CRISPR/Cas9 system on a C57BL/6J mouse background by GemPharmatech Co., Ltd (Nanjing, China). The mouse strain creation strategy involved the knockout of exon1-2 of the *Six1*-201 (ENSMUST00000050029.7) transcript region. *Wnt1-Cre* mice were obtained from the Jackson Laboratory (Bar Harbor, ME, United States). *Six1* ^*f/f*^ mice were crossed with *Six1* ^*f/+*^ *; Wnt1-cre* mice to generate *Six1* ^*f/f*^ *; Wnt1-Cre* embryos. (Supplementary Figure 1; Supplementary Table S1).

Embryos were obtained for subsequent experiments at E18.5, E16.5, and E14.5 days. The day of the appearance of a vaginal plug was defined as E0.5, and the embryos were obtained at 12:30 on each day in question. All mice were maintained and used in experiments according to the guidelines approved by the Institutional Animal Care and Use Committee (IACUC) of the Shanghai Ninth People’s Hospital affiliated to Shanghai Jiao Tong University School of Medicine.

### Skeletal preparation

Skin and soft tissue were carefully removed from E18.5 embryos and the embryos were treated in 95% ethanol overnight, followed by staining with Alcian blue for 48 h at 37°C. Embryos were washed twice with 95% ethanol for 2 h each, treated with 1% KOH for 1 h, and stained with Alizarin red for 2 h. The embryonic bone tissue was soaked in a gradient mixture of 1% KOH in glycerol (75%, 50%, 25%) and photographed.

### Histology and immunofluorescence, and TUNEL assay

The heads of the embryos were surgically isolated and fixed overnight with 4% PFA at 4°C, followed by gradient dehydration using an ethanol solution, embedded using paraffin. Hematoxylin–Eosin (HE) and Alcian blue staining were performed on 7 µm-thick paraffin sections. Immunofluorescence staining was performed with anti-Osteopontin Polyclonal antibody (22952-1-AP, Proteintech, Rosemont, IL, United States; 1:50), anti-Osterix antibody (ab209484, Abcam, Cambridge, United Kingdom; 1:200), or anti-Ki67 antibody (ab16667, Abcam; 1:100) followed by goat secondary antibody to rabbit IgG(A-11008, Thermo Fisher Scientific, Waltham, MA, United States; 1:500) following a previously described protocol (Ha et al., 2022). TUNEL staining was performed using *In Situ* Cell Death Detection Kit (11684795910, Roche, Mannheim, Germany) according to the manufacturer’s instructions. Nuclei were counterstained with 4′,6-diamidino-2-phenylindole (DAPI). Images were captured using an Olympus IX83 inverted microscope (Olympus, Tokyo, Japan).

### Cell culture, osteogenic differentiation, alkaline phosphatase (ALP) staining and cell proliferation assay

C3H10 T1/2 cells were purchased from the Cell Bank of the Chinese Academy of Sciences, Shanghai. The cells were maintained at 37°C with 5% CO_2_, and were cultured in MEM-α containing 10% FBS (10099141C, Gibco™, Thermo Fisher Scientific), 1% penicillin/streptomycin, 1% Non-Essential Amino Acids, and 1% GlutaMAX™ Supplement, and the culture medium was replaced every 2 days. Osteogenic induction medium (MUXMT-90021, Cyagen Biosciences Inc., Guangzhou, China) was used in the process of cell osteogenic differentiation (Ma et al., 2021). C3H10 T1/2 cells were seeded into 6-well plates at 50,000 cells per well and incubated at 37°C, 5% CO_2_ for 24 h. Then the regular cell culture medium was replaced with osteogenic induction medium. The osteogenic induction medium was changed every 48 h. ALP staining and RNA extraction were performed 7 days after osteogenic induction. The cells were fixed for 30 min with 4% paraformaldehyde, and ALP staining was performed using the BCIP/NBT Alkaline Phosphatase color development kit following the manufacturer’s instructions (C3206, Beyotime Biotechnology, Beijing, China). Cell proliferation was analyzed using the Cell Counting Kit-8 according to the manufacturer’s instructions (C0037, Beyotime Biotechnology). Cells were seeded in a 96-well plate at a density of 1,000 cells per well and incubated at 37°C, 5% with complete MEM-α. At 24, 48, 72, 96, and 120 h, the medium was removed and added CCK-8 solution was added to the medium and incubated for 1 h. Then, the reaction solution was read with a multimode reader (BioTek, Winooski, VT, United States) to obtain the absorbance at 450 nm.

### RNA extraction and quantitative RT-PCR

The RNA was extracted using a total RNA extraction kit (LS1040, Promega, Madison, WI, United States). Following the manufacturer’s instructions, 1 µg of total RNA was reverse transcribed into cDNA using Hifair first Strand cDNA Synthesis SuperMix (11141ES10, Yeasen Biotech, Shanghai, China) for RT-qPCR analysis. Quantitative PCR was performed on a Lightcycler 96 (Roche, Basel, Switzerland) with Hieff qPCR SYBR Green Master Mix (No Rox) (11201ES03; Yeasen Biotech). The relative expression was calculated for each gene by the 2^−ΔΔCT^ method, normalized against GAPDH expression, and presented as fold changes relative to the control. The sequences of all the primers used in this study are shown in Supplementary Table S2.

### Construction of knockdown short hairpin RNA vectors and cell infection

Short hairpin RNA (shRNA) targeting *Six1* (NM_009189.3) was designed with the following sequence: GCT​CAT​GTC​CAG​CTC​AGA​AGA. The shRNA was transfected into the pLV-shRNA-EGFP(2A) Puro vector. *Six1*-shRNA lentiviruses were packaged and amplified by co-transfecting recombinant vector together with pSPAX2 and pMD2G into 293T cells with lipo8000 and culturing for 48 h. Then the cell culture supernatants were collected and concentrated using a Universal Virus Concentration Kit (C2901M, Beyotime Institute of Biotechnology, Jiangsu, China). The virus concentrate was added to C3H10 T1/2 cells at an MOI of 50 with polybrene(12 μg/mL) and cultured for 6 h. Subsequently, the cell medium was changed and cells were cultured for a further 72 h. To obtain stably transfected cells, C3H10 T1/2 cells were cultured in MEM-α supplemented with puromycin dihydrochloride (10 μg/mL, Beyotime Institute of Biotechnology) for 7 days.

### RNA sequencing

An RNA sequencing library was prepared using a NEBNext Ultra™ RNA Library Prep Kit for Illumina and was sequenced on an Illumina novaseq6000. Differentially-expressed genes (DEGs) were determined with log2 expression fold change(log2FC) > 1 and a *p*-value (padj) < 0.05. Gene Ontology (GO) enrichment analysis of DEGs was performed using the clusterProfiler package in R.

### Cleavage under targets and tagmentation (CUT&Tag) library preparation

Cleavage under targets and tagmentation (CUT&Tag) libraries were prepared using an *In-Situ* ChIP Library Prep Kit (TD901, Vazyme Biotech, Nanjing, China). Adherent cultured cells were digested with 0.25% trypsin, and 50,000 C3H10 T1/2 cells per sample were used in two biological replicates. After centrifugation at 600 *g* for 5 min at room temperature, the cells were washed twice with Wash Buffer. Cells were bound to conA beads and incubated with anti-Six1 antibody (#12891, CST, Danvers, MA, United States; 1:50) overnight at 4°C. The primary antibody was removed, and then a secondary antibody (Goat Anti-Rabbit IgG, Vazyme) was diluted (1:100) in DIG Wash buffer and incubated with cells at room temperature in a shaker for 1 h. Next, cells were incubated with pA-Tn5 transposon complex (0.04 µM) at room temperature for 1 h. DNA was extracted and then purified using Hieff NGS^®^ DNA Selection Beads (12601ES03, Yeasen Biotech). The libraries were sequenced on the Novaseq-150PE platform and 150-bp pair-end were generated. The sequencing depth was 10G base pair raw data.

### Statistical analysis

GraphPad Prism v.9.0 for Windows (GraphPad Software, LaJolla, CA, United States) was used for statistical analysis. All numerical data are presented as means ± SD. Independent two-tailed Student’s *t*-tests were used for comparisons between two groups, and differences were considered statistically significant at a *p*-value *<* 0.05.

## Results

### The *Six1* knockout mice exhibited craniofacial deformity

**FIGURE 1:**
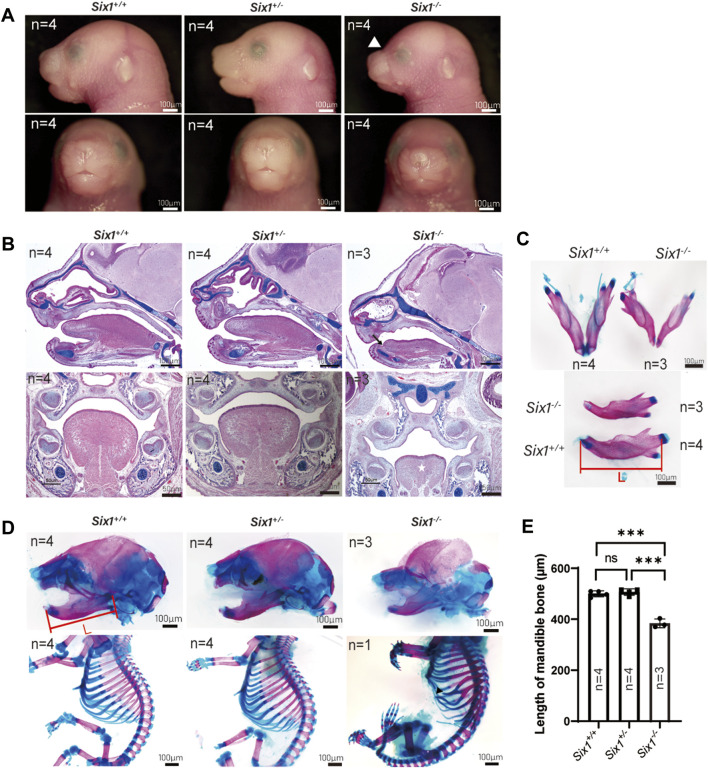
The *Six1* deletion mice resulted in craniofacial deformity. **(A)** Lateral view (top) and frontal (bottom) gross morphology of E18.5 heads of the *Six1* ^*−/−*^, *Six1* ± and *Six1* ^*+/+*^ embryos. *Six1* ^*−/−*^ embryos exhibit short mandible and classical abnormal curve between nose and forehead (white arrowhead). **(B)** Hematoxylin and eosin (HE) and Alcian blue staining of sagittal sections (top) and frontal (bottom) sections of *Six1* ^*−/−*^, *Six1* ^*+/−*^ and *Six1* ^*+/+*^ embryo at E16.5. *Six1* ^*−/−*^ embryos display a short mandible, ankyloglossia (black arrow), and uvula deformity (white star). **(C)** Skeletal staining of E18.5 mandible of the *Six1* ^*−/−*^ and *Six1* ^*+/+*^ embryo. *Six1* ^*−/−*^ mice exhibit a shortened mandible. L: length of mandible. **(D)** Skeletal alizarin red and Alcian blue staining of E18.5 heads (top) and body (bottom) of the *Six1* ^*−/−*^, *Six1* ^*+/−*^ and *Six1* ^*+/+*^ embryo. The black arrowhead points to bifurcated ribs. **(E)** Quantification of the mandibular length from *Six1* ^*−/−*^, *Six1* ^*+/−*^ and *Six1* ^*+/+*^ embryos at E18.5.

To explore the role of Six1 in craniofacial development, we generated *Six1* knockout mice using the CRISPR/Cas9-based approach. To ensure the efficiency of Six1 knockout in *Six1* ^*−/−*^ mice, we examined the RNA-seq data at E18.5 and verified the *Six1* expression level by RT-qPCR (Supplementary Figure S5). By comparing gross images and skeletal staining of *Six1* ^*+/+*^ (n = 4) and *Six1* ^*+/−*^ (n = 4) embryos at E18.5, we found that the heterozygous embryos had no craniofacial deformities and no differences in mandibular length, and that heterozygous mice survived and reproduced normally (Figures 1A–D). Hence, we used *Six1* ^*+/−*^ to mate with each other to obtain *Six1* ^*−/−*^ pups, and the *Six1* ^*−/−*^ birth probability conformed to the Mendelian ratio (14/55). All the *Six1* ^*−/−*^ mice that died at birth exhibited a wide range of craniofacial deformities, including microsomia, high arched palate, and a small tongue. Morphologic observation and examination of skeletal preparations at E18.5 revealed that the mandible length of *Six1* ^*−/−*^ mice (n = 3) was significantly shorter than wild-type or heterozygous littermates. Analyses of HE staining of E16.5 embryos revealed that *Six1* ^*−/−*^ (n = 3) mice exhibited a high palate and small tongue with ankyloglossia (Figure 1B). The volume of tongue muscle of *Six1* ^*−/−*^ mice was significantly reduced. We also found that mice (1/3) exhibited bifurcated ribs, characterized by abnormal fusion between the upper and lower rib cartilage (Figure 1D). The mandibular length of *Six1* ^*−/−*^ (n = 3) embryos at E18.5 was significantly shorter than that of *Six1* ^*+/+*^(n = 4) (*p* < 0.0001) (Figures 1C, E). *Six1* knockout mice exhibited a stable phenotype of a short jaw with 100% penetrance (14/14). The craniofacial phenotype of Six1 knockout mice was similar to that reported in the literature (Liu et al., 2019), and the skeletal deformities were also as reported in the literature (Li et al., 2003). These results demonstrate that *Six1* knockout mice were successfully constructed, and this model is suitable for studying the causes of the short mandible.

### Conditional knockout of *Six1* in cranial neural crest cells caused microsomia and cleft palate

**FIGURE 2:**
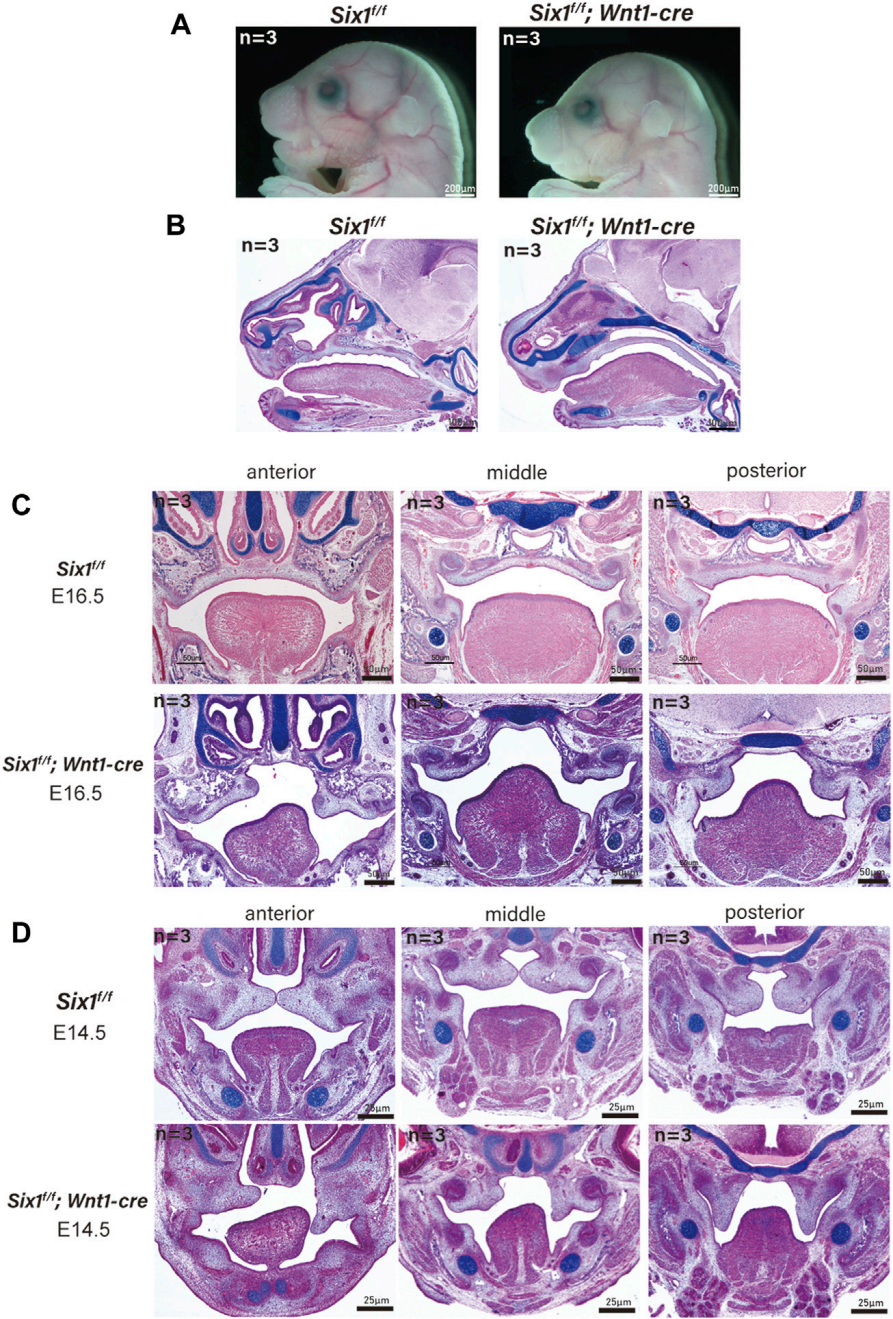
The conditional knockout of *Six1* in cranial neural crest cells (CNCCs) results in microsomia. **(A)** Gross morphology of E18.5 heads of the *Six1* ^*f/f*^ *; Wnt1-Cre* and *Six1* ^*f/f*^ embryos*.* **(B)** HE and Alcian blue staining of sagittal sections showing the morphology of mandible of the *Six1* ^*f/f*^ *; Wnt1-Cre* and *Six1* ^*f/f*^ embryos at E18.5. *Six1* ^*f/f*^ *; Wnt1-Cre* embryo shows a short mandible. **(C, D)** HE and Alcian blue staining of frontal sections of the *Six1* ^*f/f*^ *; Wnt1-Cre* and *Six1* ^*f/f*^ embryos at E16.5 **(C)** and E14.5 **(D)**. *Six1* ^*f/f*^ *; Wnt1-Cre* embryo shows cleft palate at E16.5 and E14.5.

The mandible is derived from neural crest cell-derived tissues (Parada and Chai, 2015). To explicitly assess whether the craniofacial defects were caused by the loss of *Six1* function in CNCC-derived ectomesenchyme, we generated *Six1* ^*f/f*^ mice and crossed them with *Wnt1-Cre* mice to conditionally knockout *Six1* in CNCCs (*Six1* ^*f/f*^ *; Wnt1-Cre*). *Wnt1-Cre* pups were born in accordance with the Mendelian ratio. However, the majority of *Six1* ^*f/f*^ *; Wnt1-Cre* pups died at birth. By morphological analysis and examination of skeletal preparations at E18.5, we found that all *Six1* ^*f/f*^ *; Wnt1-Cre* (n = 3) pups exhibited microsomia compared with control littermates, and the phenotype was similar to that of *Six1* knockout mice (Figure 2A).

Interestingly, a new phenotype, cleft palate (70%, 7/10) with ankyloglossia, was found in *Six1* ^*f/f*^ *; Wnt1-Cre* mice without atrophy of tongue muscle. We further studied the *Six1* ^*f/f*^ *; Wnt1-Cre* mice at E16.5 (n = 3) and E14.5 (n = 3), and found that the tongue muscle occupied the development space of the palate at E14.5, which prevented palatal lifting and led to the development of cleft palate (Figures 2C, D). Tongue connective tissue is derived from CNCCs, whereas the skeletal muscles originate from the myoblasts (Noden and Francis-West, 2006). The reduction in oral volume was due to the lack of mandibular development, while the unaffected tongue muscle volume resulted in an increased tongue muscle height. Therefore, the cleft palate phenotype of conditional knockout *Six1* mice might be a secondary cleft palate. These data indicate that Six1 plays a crucial role in the growth and differentiation of CNCC-derived mesenchyme during craniofacial development.

### *Six1* knockout resulted in decreased mandibular bone formation and altered gene expression in mice

**FIGURE 3:**
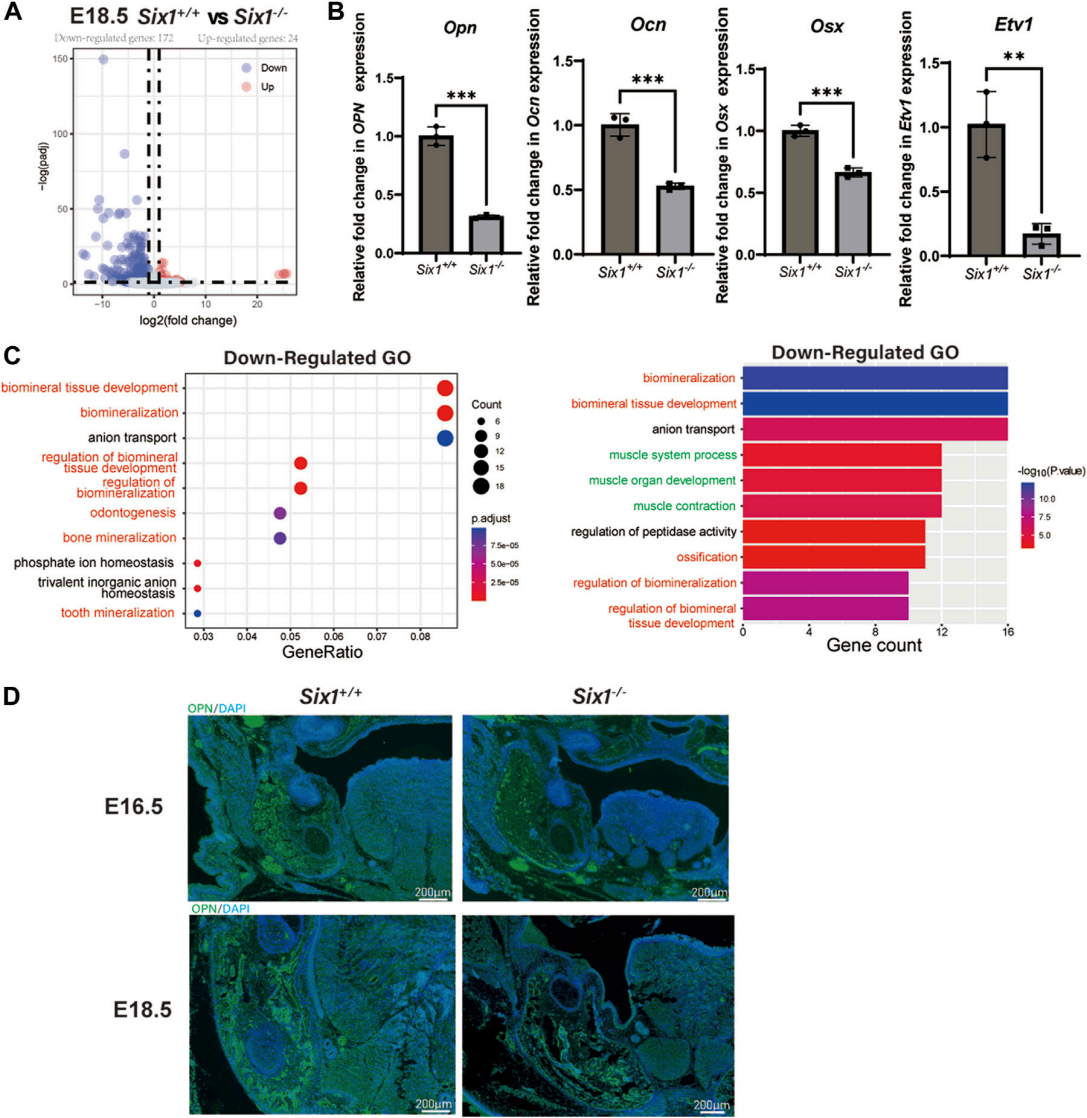
RNA-seq of the mandibular tissue from E18.5 *Six1* ^*−/−*^ and *Six1* ^*+/+*^ embryos. **(A)** Volcano plots show differentially expressed genes between *Six1* ^*−/−*^ and *Six1* ^*+/+*^ mandibular samples. **(B)** RT-qPCR analysis of *Opn*, *Ocn, Osx* and *Etv1* in *Six1* ^*−/−*^ and *Six1* ^*+/+*^ mandibular tissues at E18.5. **(C)** GO enrichment analysis of genes significantly downregulated in *Six1* ^*−/−*^ mandible. **(D)** Immunofluorescence staining of OPN in mandible of *Six1* ^*−/−*^ and *Six1* ^*+/+*^ embryos at E16.5 and E18.5.

We reasoned that *Six1* deletion might disrupt the complex gene expression pattern during craniofacial development. To reveal the key genes regulated by the transcription factor Six1 during mandibular development, we surgically isolated mandibular skeletal tissues and surrounding soft tissues from E18.5 *Six1* ^*−/−*^ or littermate control wild-type *Six1* ^*+/+*^ mice and performed bulk RNA-seq on two independent biological replicates for each genotype. Analysis of the RNA-seq data revealed that the *Six1* transcripts were completely absent in *Six1* ^*−/−*^. Comparing the results of *Six1* ^*−/−*^ and *Six1* ^*+/+*^ mice revealed that 196 genes exhibited significant expression changes (log2FC > 1, padj < 0.05). Among these, 172 genes were downregulated, and 24 genes were upregulated (Figure 3A; Supplementary Table S1). Correlation analysis of RNA-seq showed that *Six1* ^*−/−*^ and *Six1* ^*+/+*^ were significantly different (Supplementary Figure S2). The uniquely mapped reads were all greater than 85%, indicating high-quality sequencing data (Supplementary Table S3). Notably, *Six1* ^*−/−*^ showed significantly downregulated expression of osteogenic and mineralization genes at E18.5, including *Opn*, *Ocn,* and *Osx* (Figure 3B). GO enrichment analysis of downregulated DEGs showed that multiple development-related biological processes were impacted, and the DEGs were significantly enriched in “ossification”, “biomineralization”, and “biomineral tissue development” (Figure 3C; Supplementary Table S2). Using immunofluorescence staining, we further verified that the level of Opn in the *Six1* ^*−/−*^ mandible was significantly lower than that in heterozygous littermates at E16.5 and E18.5 (Figure 3D). We also found a moderate downregulation in the mandibular region of *Six1* ^*−/−*^ mice by Osx immunofluorescence staining, which was consistent with the RT-qPCR results (Supplementary Figure S3). *Six1* ^*−/−*^ knockout mice had no significant effect on the proliferation and apoptosis of the mandible at E16.5 (Supplementary Figure S4). Collectively, these data suggest that the knockout of Six1 impaired mandibular bone formation by regulating the expression of critical genes involved in osteogenesis.

Interestingly, we also found that genes related to muscle development were significantly downregulated (Figure 3C). Observing the downregulated GO term “muscle organ development” revealed that their enriched genes include *Etv1*(Tenney et al., 2019), *Tcap* (Markert et al., 2010), *Lbx1*(Wang et al., 2022), *Actn3* (Nicot et al., 2021), and *Fos* (Almada et al., 2021), which could explain the uvula deformity observed in *Six1* ^*−/−*^ mice. RT-qPCR showed that *Etv1* expression was significantly reduced in the mandibular tissues of *Six1* ^*−/−*^ mice (Figure 3B). These results suggest that the craniofacial defects observed in *Six1* ^*−/−*^ mice result from profound dysregulation of genes related to skeletal and muscle development.

### *Six1* knockdown decreased the osteogenic differentiation capacity of C3H10 T1/2 cells

**FIGURE 4:**
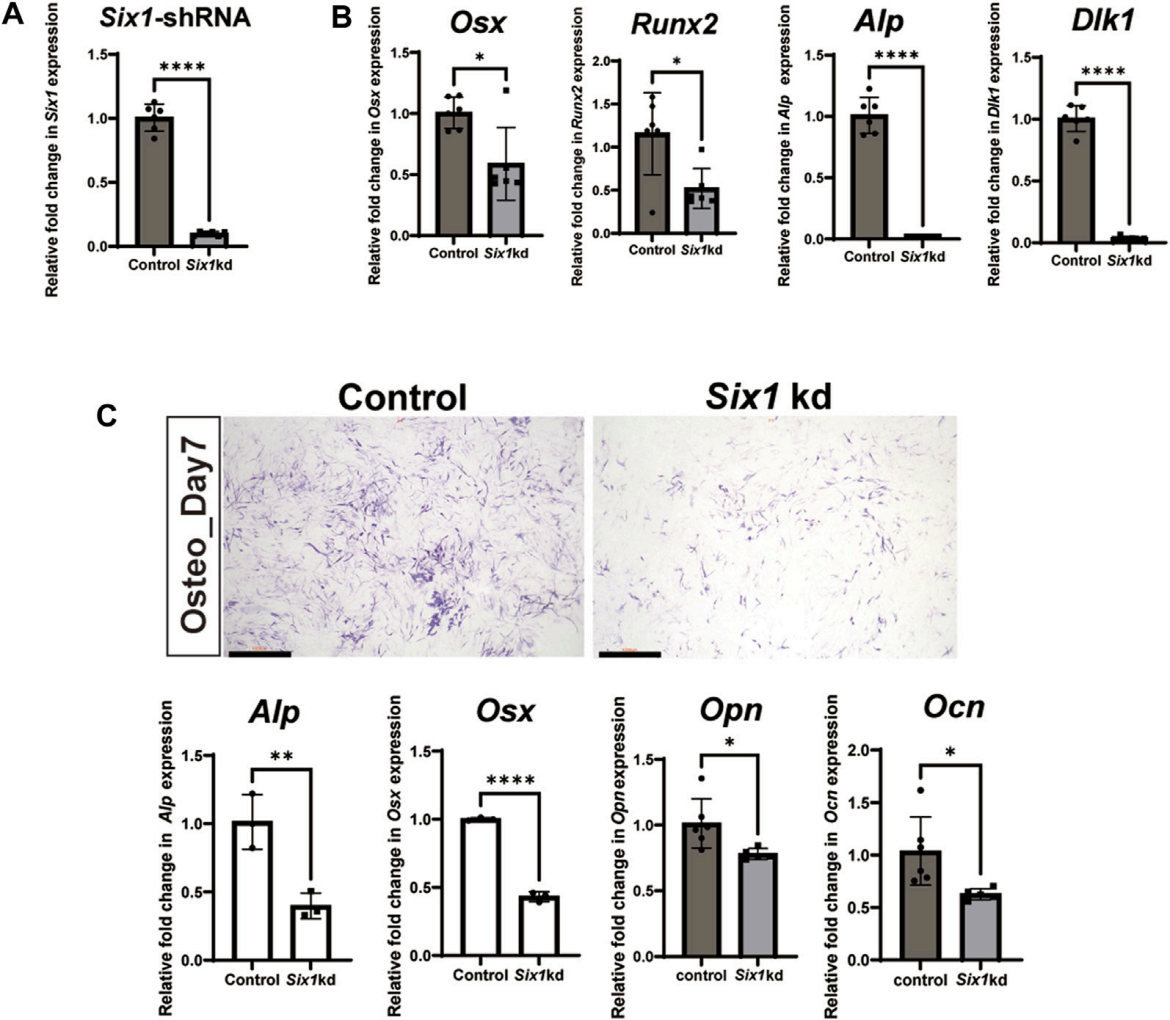
The effect of *Six1* on the osteogenic differentiation of C3H10 T1/2 cells. **(A)** RT-qPCR analysis of C3H10 T1/2 cells transfected with negative control (Control) or *Six1* knockdown cells (*Six1*kd) (n = 6). **(B)** RT-qPCR analysis of *Osx*, *Runx2*, *Alp*, and *Dlk1* in control and *Six1* knockdown cells (*Six1*kd). **(C)** Alkaline phosphatase (ALP) staining of negative control C3H10 T1/2 cells (control) and *Six1*kd cells after osteogenic induction for 7 days. RT-qPCR analysis of *Alp*, *Osx*, *Opn*, and *Ocn* after osteogenic induction for 7 days of control and *Six1*kd cells. Scale bar in C, 1000 µm.

To further explore the role of *Six1* during mandibular osteogenesis, we performed osteogenic induction assay on the mouse embryonic mesenchymal stem cell line (C3H10 T1/2) to investigate the potential mechanisms *in vitro*. By performing RT-qPCR, we showed that the expression of *Six1* could be readily detected in C3H10 T1/2 cells (Figure 4A). We then performed *Six1* knockdown by infecting C3H10 T1/2 cells with lentivirus expressing an shRNA specifically targeting *Six1*, and verified that the *Six1* mRNA was markedly depleted in *Six1* knockdown cells (*p* < 0.0001). RT-qPCR analysis showed that several critical osteogenic genes, including *Osx*, *Runx2*, *Alp*, and *Dlk1,* were downregulated in *Six1* knockdown cells (Figure 4B). We further compared the osteogenic differentiation capacity of control and *Six1* knockdown C3H10 T1/2 cells after osteogenic induction for 7 days by quantifying alkaline phosphatase (ALP) staining as well as measuring the mRNA levels of *Alp*, *Osx*, *Opn*, and *Ocn* by RT-qPCR (Figure 4C). *Six1* knockdown C3H10 T1/2 cells exhibited lower ALP activity and downregulation of *Osx*, *Opn*, and *Ocn* expression. The proliferation activity of *Six1* knockdown C3H10 T1/2 cells was inhibited (Supplementary Figure S4). These results indicate that *Six1* knockdown leads to the decline of osteogenic marker genes expression and reduced osteogenic differentiation in osteogenesis.

### *Six1* promotes osteogenic function by regulating multiple osteogenesis-related genes

**FIGURE 5:**
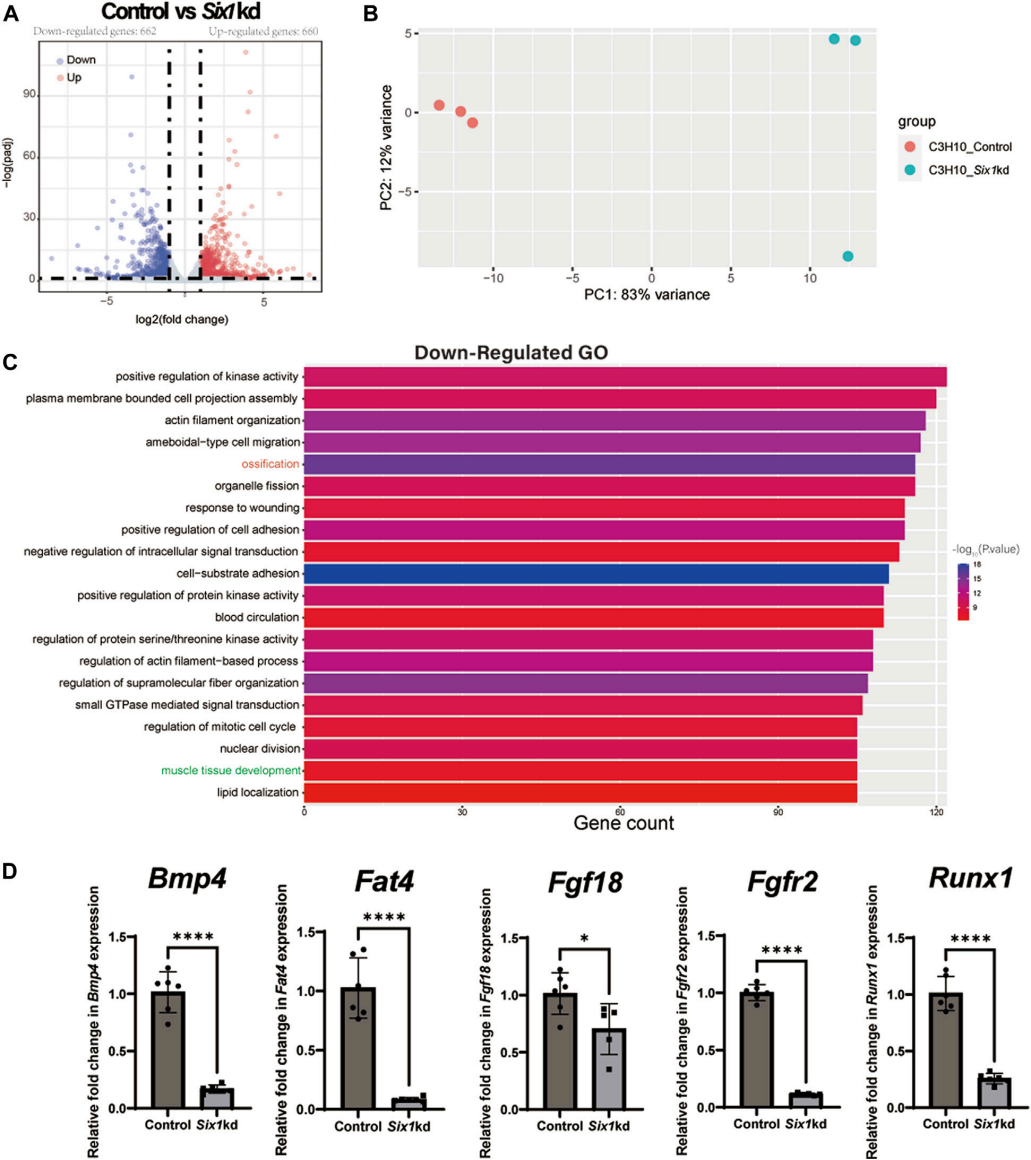
RNA-seq of C3H10 T1/2 cells transfected with negative control (Control) or *Six1-*shRNAs (*Six1*kd) lentivirus (n = 3). **(A)** Volcano plots for all the genes of the control and *Six1*kd groups. Dots on both sides indicate up and downregulated differentially expressed genes (DEGs; p-adj < 0.05). **(B)** PCA plot showing the correlation between RNA-seq replicates. **(C)** The terms associated with biological processes (p-adj < 0.05) involving the downregulated genes in the *Six1*kd group. **(D)** RT-qPCR analysis of *Bmp4*, *Fat4*, *Fgf18*, *Fgfr2*, and *Runx1* in control and *Six1*kd cells.

To investigate the underlying mechanism by which Six1 regulates osteogenic differentiation of C3H10 T1/2 cells, we analyzed the transcriptional effect of *Six1* knockdown on C3H10 T1/2 cells by performing RNA sequencing on three biological replicates of control and *Six1* knockdown cells. The knockdown and control groups showed a more significant correlation with each other, indicating good quality and repeatability of the RNA sequencing dataset (Figure 5B, Supplementary Figure S2). Analysis of the DEGs (log2FC > 1, padj<0.05) revealed that 662 genes were downregulated and 660 genes were upregulated in *Six1* knockdown cells compared with that in control cells (Figure 5A; Supplementary Table S3). GO analysis of the downregulated DEGs showed that the knockdown of *Six1* suppressed osteogenic differentiation through the regulation of biological processes associated with “ossification” and “muscle tissue development” (Figure 5C; Supplementary Table S4). We further validated the mRNA expression of several osteogenic differentiation related genes in *Six1* knockdown C3H10 T1/2 cells. Consistent with the RNA-Seq results, the mRNA expression of *Bmp4*, *Fat4*, *Fgf18*, *Fgfr2*, and *Runx1* significantly decreased (Figure 5D).

### SIX1 directly binds to the promoters of *Bmp4, Fgfr2, Fgf18*, and *Fat4* and regulates their expression

**FIGURE 6:**
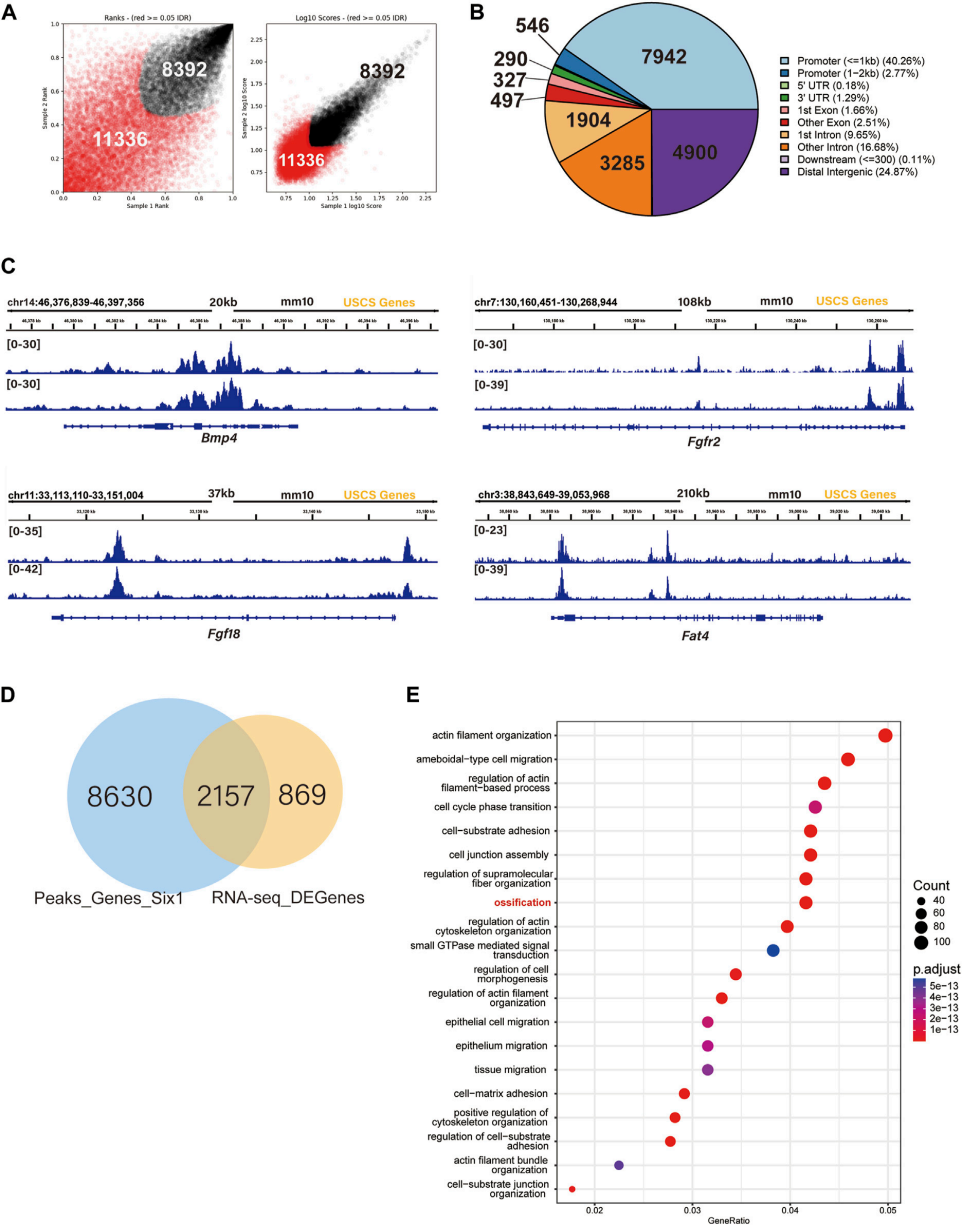
SIX1 directly regulates the promoter of osteogenic differentiation-related genes. **(A)** IDR tests the peaks of two biological replication. **(B)** Genomic distribution of *Six1*-enriched regions. **(C)** Six1 directly binds the promoter of *Bmp4*, *Fgfr2*, *Fgf18*, and *Fat4*. **(D)** A Venn diagram indicating overlap of Six1-binding genes and RNA-seq DEGs. **(E)** GO enrichment analysis of shared genes between Six1-binding genes and RNA-seq DEGs.

To further explore the mechanism by which Six1 regulates osteogenesis, we examined the genome-wide occupancy of Six1 in C3H10 T1/2 cells by performing CUT&Tag. The IDR consistency test was performed on the two sets of CUT&Tag data, and a total of 19,728 peaks were obtained (Figure 6A; Supplementary Table S5). Among the Six1 peaks, 40.26% were located in the promoter region (≤ 1 kb from the TSS), while 24.87% were located in the distal intergenic region (Figure 6B). These CUT&Tag peaks were annotated to the 10,788 closest genes. In addition, CUT&Tag assay showed that Six1 directly regulated the promoters of *Bmp4*, *Fgfr2*, *Fgf18*, and *Fat4*, all of which have been reported to play important roles in osteogenesis and were downregulated in *Six1* knockdown C3H10 T1/2 cells (Figure 6C). Importantly, nearly 3/4 (2,157/3,027) of the DEGs from RNA-seq were associated with the Six1 peaks (Figure 6D). GO enrichment analysis of these 2,157 Six1*-*bound DEGs again showed that the ossification function was significantly enriched (Figure 6E). Six1 was also showed to bind to the promoter of *Etv1*, a gene involved in muscle development (Tenney et al., 2019). Taken together, our data strongly suggest that Six1 regulates the expression of a group of genes involved in bone and muscle development by binding to their promoters or cis-regulatory regions, thereby influencing craniofacial development and morphogenesis.

## Discussion

Six1 plays an important role in embryonic development and is one of the pathogenic genes of human Branchio-oto-renal syndrome (BOR) (Shah et al., 2020). Children with BOR show hearing loss, renal abnormalities, and microsomia(Kochhar et al., 2007). Six1 is widely expressed in the mesenchymal and sensory epithelium of the craniofacial region (Liu et al., 2019; Li et al., 2020). Studies have revealed that Six1 regulates auditory sensory epithelial differentiation, and participates in ear development (Li et al., 2020). For craniofacial development, *Six1*-null mice exhibit abnormal craniofacial skeletal development, including microsomia and the formation of a novel bone in the zygomatic arch (Tavares et al., 2017; Liu et al., 2019). However, the mechanisms of *Six1* during mandibular development remain unclear.

Tavares et al. found that *Six1* ^*−/−*^ mice upregulated *Edn1* signaling in the first and second branchial epithelium, while Six1 was expressed in the adjacent mesenchymal region, suggesting that Six1 may participate in craniofacial development through epithelial-mesenchymal interaction (Tavares et al., 2017). We demonstrated that the conditional knockout of *Six1* in mesenchyme largely phenocopied the underdevelopment of the mandible observed in *Six1* ^*−/−*^ mice, thus demonstrating that Six1 regulates development of the mandible in the ectodermal mesenchyme. Interestingly, *Six1* ^*f/f*^ *; Wnt1-Cre* mice showed normal tongue muscle but the cleft palate, a more severe craniofacial deformity. Tongue muscle originates from mesodermal myoblasts, and CNCC-derived mesenchyme in tongue development acts as a scaffold for the organization of migrating myoblasts into the myogenic core (Parada and Chai, 2015). Hence, *Wnt1-cre* does not knockout *Six1* in the tongue muscle, but specifically knockout *Six1* in the mandible. *Six1* ^*f/f*^ *; Wnt1-Cre* mice exhibited no tongue abnormalities, but showed a lack of Six1 expression in the mandible, resulting in reduced oral volume. We surmise that when the palate begins to fuse at E14.5, the insufficient oral volume in *Six1* ^*f/f*^ *; Wnt1-Cre* mice may cause the tongue to occupy the palatal space, thereby affecting the palatal lift and eventually leading to secondary cleft palate.

We found that the expression of osteogenesis-related genes, such as *Opn*, *Ocn* and *Osx*, was significantly downregulated in the mandible of *Six1* ^*−/−*^ mice at E18.5, suggesting that Six1 may regulate multiple osteogenesis-related genes. It was previously reported that *Six1* ^*−/−*^ mice showed increased *Osx* expression in the maxillary and hinge region, and zygomatic process hyperplasia which developed into a thicker rod-shaped bone (Tavares et al., 2017). However, in our study, *Six1* ^*−/−*^ mice showed reduced *Osx* expression in the mandible and defects in mandibular osteogenesis. Six1 does not affect the proliferation and apoptosis of mandibular development at the late stages of embryonic development. We propose that Six1 regulates different signaling pathways in the maxilla and mandible, thus producing different biological effects. More studies are needed further to explore the mechanism of Six1 during craniofacial skeletal development.

Our analyses of C3H10 T1/2 cells and mandibular tissue RNA-seq indicate that Six1 regulates the expression of multiple osteogenesis-related genes. The spatiotemporal expression of Bmp4 highly coincides with that of Six1, and it directly regulates the expression of *Msx1* and other genes in the BMP family, and plays an important role in the process of mandibular osteogenesis(Xu et al., 2021). *Bmp4* ^*f/f*^ *; Wnt1-Cre* mutant pups exhibited short mandible (Xu et al., 2021). Similar phenotypes were observed in *Fgf18* ^−/−^ embryos (Hung et al., 2016). In addition, mice with deletion of *Fgf18* in neural crest cells also exhibited a shortened mandible, suggesting that *Six1* and *Fgf18* in neural crest mesenchymal cells may be jointly involved in mandibular osteogenesis (Yue et al., 2021). Low expression of *Fgfr2* is also closely related to cells’ decreased osteogenic ability (Jiang et al., 2019). The *Dchs1-Fat4* signaling pathway is involved in the process of osteoblast differentiation in the mouse mandible and skull and plays a positive role in early *Runx2* progenitors (Mao et al., 2016; Crespo-Enriquez et al., 2019). Our data suggest that Six1 regulating mandible development at least in part through regulating downstream genes *Fgfr2*, *Fgf18*, *Bmp4*, and *Fat4*. Future *in vivo* studies will shed more light on how Six1 coordinates the spatiotemporal expression of these genes to achieve proper craniofacial skeletal formation.

CUT&Tag assay showed that nearly half of the Six1 binding sites were located near the promoter of the downstream gene. Our results demonstrated that the changes in gene expression induced by Six1 knockdown were largely due to the direct regulation of Six1 on its downstream genes. For example, Six1 directly binds to the promoters of *Fgfr2*, *Fgf18*, *Bmp4*, and *Fat4* and regulates their transcription. Interestingly, our results also showed that a significant fraction of Six1 peaks are located in the intergenic regions, which likely correspond to cis-regulatory elements such as enhancers. Increasing evidence suggests that the enhancers play critical roles in orchestrating the precise gene expression patterns during craniofacial development (Attanasio et al., 2013). Future investigation on these Six1-bound enhancers may open new avenues for studying the functions of Six1 in craniofacial development and abnormality.

## Conclusion

In conclusion, our findings suggest that the transcription factor Six1 is critical for mandible development. Our *Six1* knockout and conditional knockout mouse models provide valuable animal models for future studies of skeletal development during craniofacial development. By integrating RNA-Seq and CUT&Tag, we identified potential target genes of Six1 that are involved in osteogenic differentiation. Future studies building on these findings will further elucidate the mechanisms by which Six1 regulates mandibular osteogenesis during embryonic development.

## Data Availability

The data presented in the study are deposited in the National Center for Biotechnology Information (NCBI) Gene Expression Omnibus (GEO), accession number GSE216761.
