# Supplementary material for: Ectomesenchymal Six1 controls mandibular skeleton formation
Source: Front Genet. 2023 Feb 8;14:1082911. doi: 10.3389/fgene.2023.1082911 (PMC9946248; doi:10.3389/fgene.2023.1082911)
Supplement: Supplementary file 3 [file Presentation1.pdf]

Supplementary Material

1 Supplementary Figures

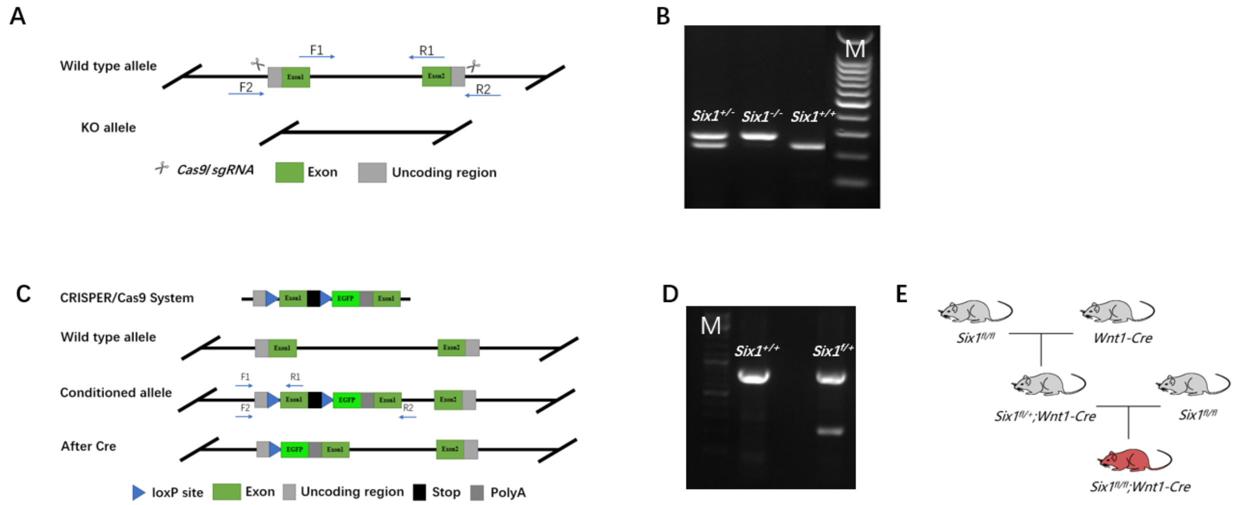

Supplementary Figure 1. (**A**) Schematic diagram of the generation of *Six1* knockout mice. (**B**) Genotyping of *Six1* knockout mice. *Six1*$^{-/-}$ mice have a 298bp band, *Six1*$^{+/+}$ mice have a 254bp band, and *Six1*$^{+/-}$ mice have both 298bp and 254bp bands. (**C**) Schematic diagram of the generation of *Six1* conditional knockout mice. (**D**) Genotyping of *Six1* conditional knockout mice. *Six1*$^{+/+}$ mice have a 803bp band, and *Six1*$^{f/+}$ mice have both 803bp and 223bp bands. (**E**) Schematic diagram of the *Six1*$^{f/f}$*; Wnt1-Cre* mouse construction strategy. M, Marker.

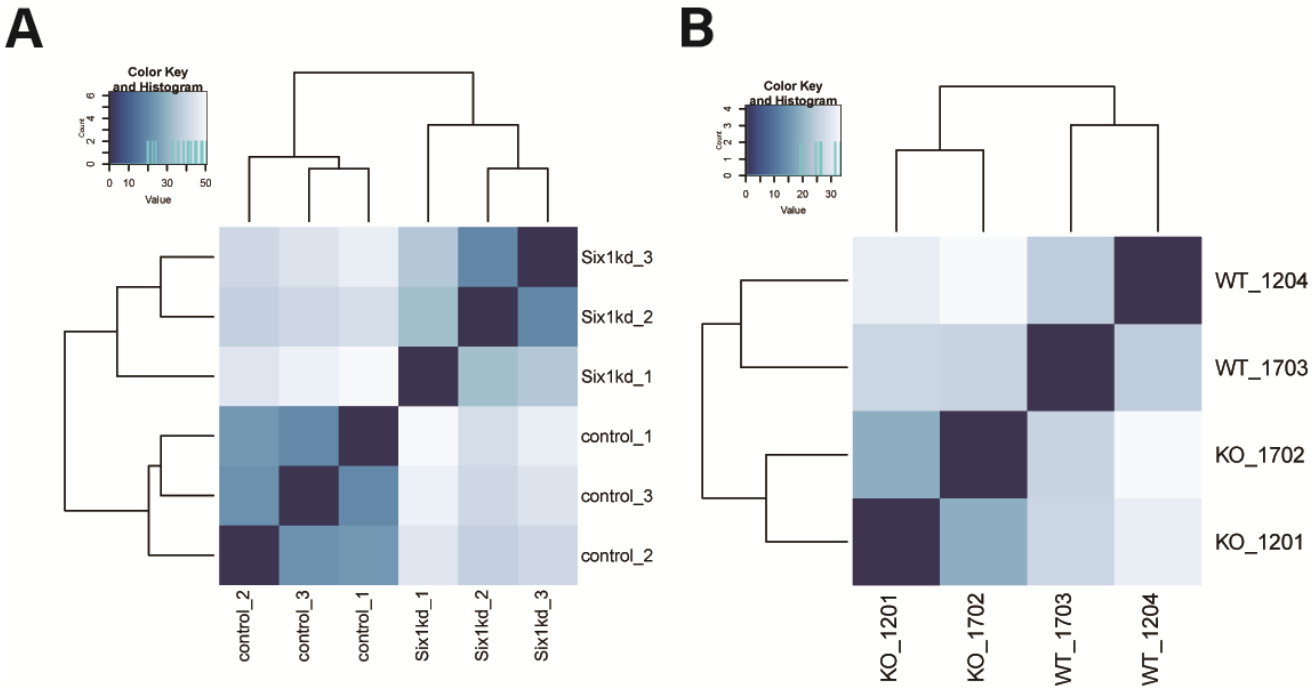

Supplementary Figure 2. (**A**) Correlation matrix for RNA-sequencing of *Six1* knockdown and control C3H10 T1/2 cells. (**B**) Correlation matrix for RNA-sequencing of *Six1*$^{-/-}$ and *Six1*$^{+/+}$ embryos at E18.5.

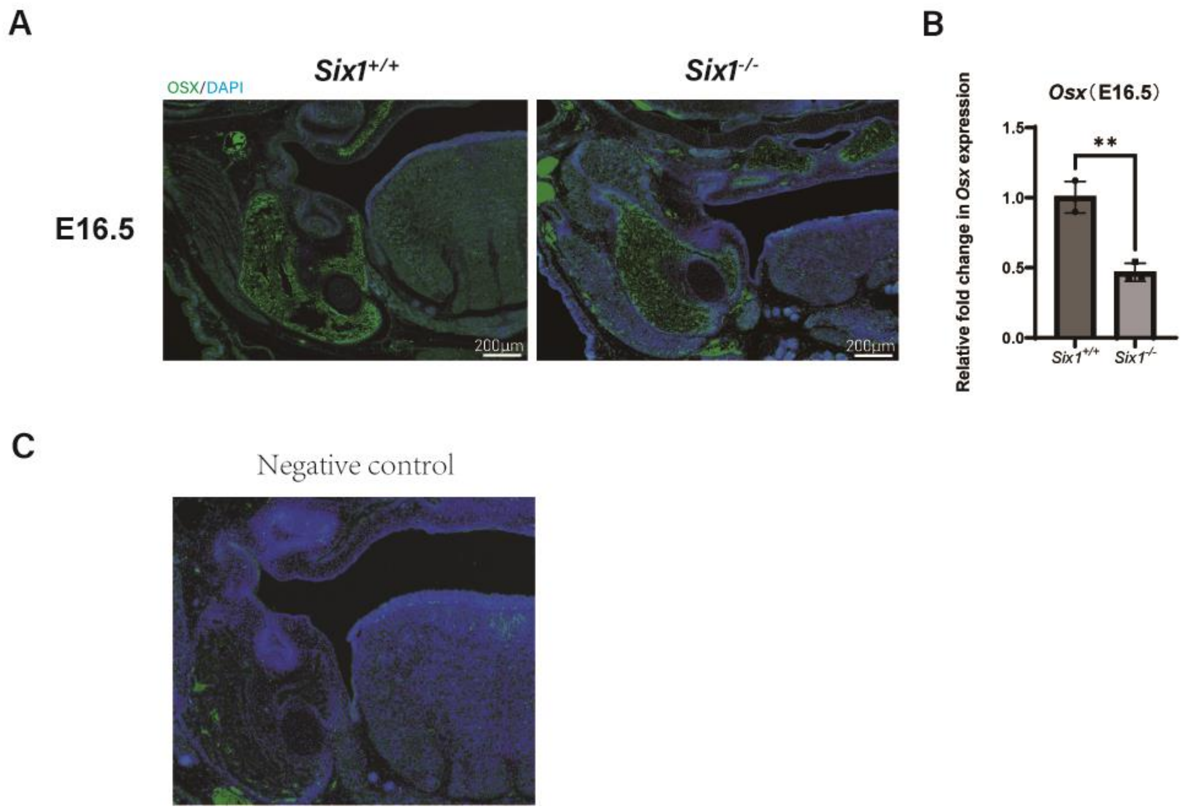

Supplementary Figure 3. (**A**) Immunofluorescence staining of Osx in mandible of *Six1*$^{-/-}$ and *Six1*$^{+/+}$ embryos at E16.5. (**B**) RT-qPCR analysis of *Osx* in *Six1*$^{-/-}$ and *Six1*$^{+/+}$ mandibular tissues at E16.5. (**C**) Negative controls of the immunofluorescence staining.

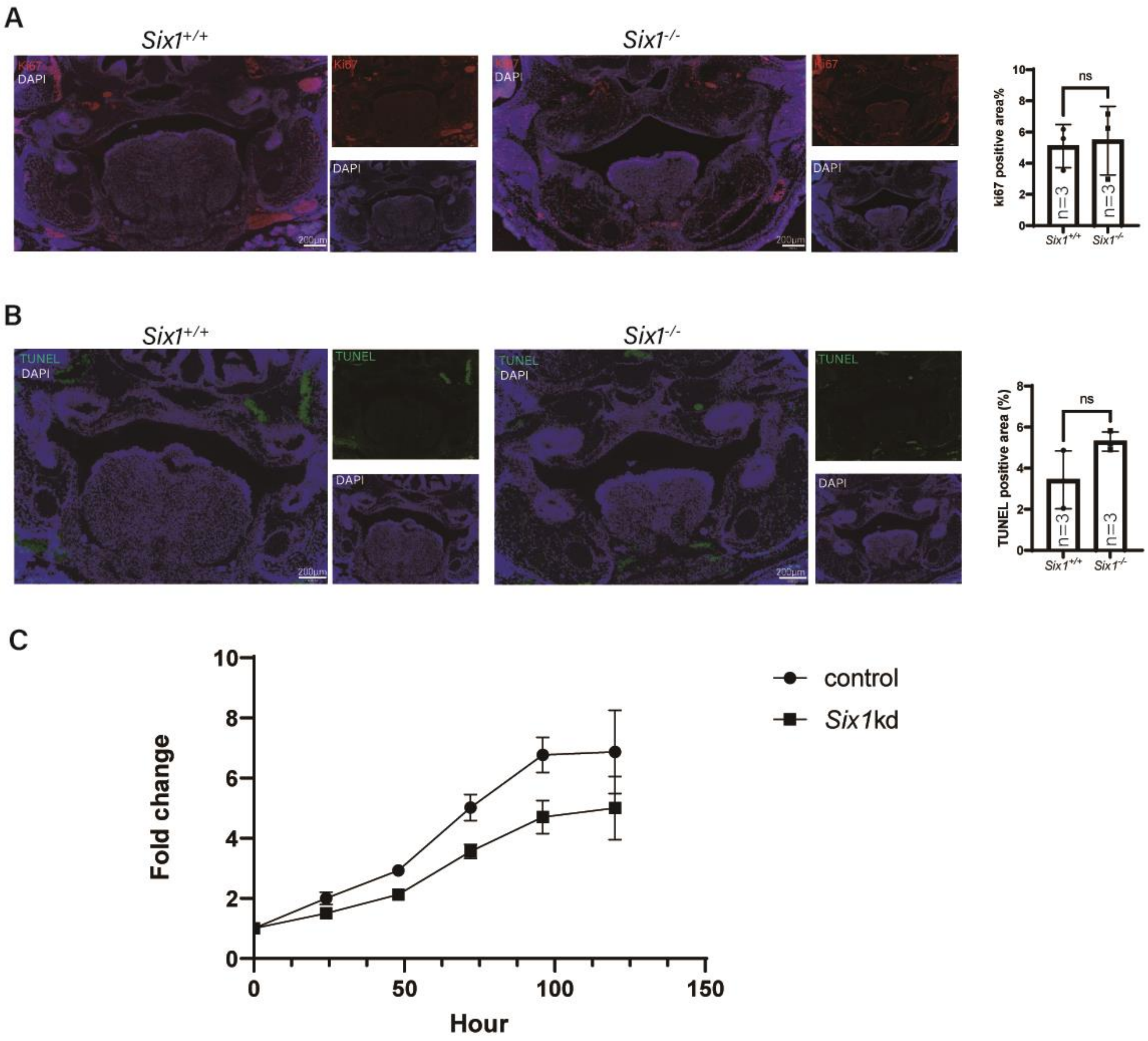

Supplementary Figure 4. (**A**) Immunofluorescence staining of Ki67 in mandible of *Six1*$^{-/-}$ and *Six1*$^{+/+}$ embryos at E16.5, and the statistical analysis of Ki-67 positive area. (**B**) TUNEL assay enotyping of *Six1*$^{-/-}$ and *Six1*$^{+/+}$ embryos at E16.5, and the statistical analysis of TUNEL positive area. (**C**) Cell proliferation assays were performed for control and Six1 knockdown C3H10 T1/2 cells at 0, 24, 48, 72, 96, or 120 hours (0–5 days).

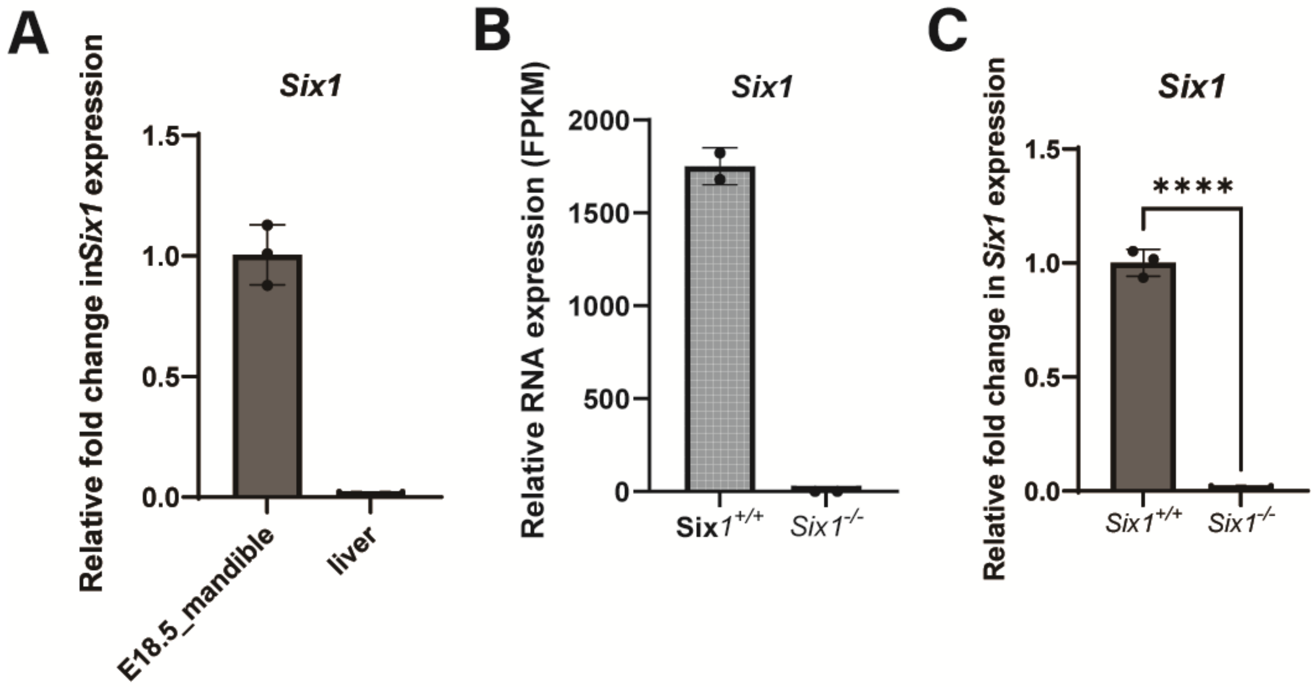

Supplementary Figure 5. (A) RT-qPCR analysis of *Six1* in mandible and liver of E18.5 mice. (B) Bar graphs showing the Six1 expression levels in Six1$^{-/-}$ and Six1$^{+/+}$ mandibular tissue according to RNA-seq. (C) RT-qPCR analysis of *Six1* in Six1$^{-/-}$ and Six1$^{+/+}$ mandibular tissue at E18.5.

2 Supplementary tables

TABLE 1 List of primer sets used for genotyping.

**Mouse model**		**Primer (5'-3')**
***Six1* knockout mice**	F1:	GCCTCTGCACACCAGAGATAGG
	R1:	GAATACGCACATTCTGGATCGG
	F2:	TTGGGTTTGTTTCATGCCCTC
	R2:	CGAAATGAAGCAGAACTCTCCTCC
***Six1* conditional knockout mice**	F1:	ACTTCTCTTCGTCTTCCTTGGCC
	R1:	GGCCCGATAACTTCGTATAGCATAC
	F2:	ACTTCTCTTCGTCTTCCTTGGCC
	R2:	AAGGGAGCGAAGGGTGAAACTT
**Wnt1-cre**	F1:	ATTTGCCTGCATTACCGGTCG
	R1:	CAGCATTGCTGTCACTTGGTC

TABLE 2 List of primer sets used for RT-qPCR.

Genes	Forward (5'-3')	Reverse (5'-3')
Alp	CGGGACTGGTACTCGGATAA	ATTCCACGTCGGTTCTGTTC
Osx	CCTTCCCTCACTCATTTCCTGG	TGTTGCCTGGACCTGGTGAGAT
Oc**n**	GAACAGACAAGTCCCACACAG	GAGCTGCTGTGACATCCATAC
Opn	AGCAAGAAACTCTTCCAAGCAA	GTGAGATTCGTCAGATTCATCCG
Etv1	TTAAGTGCAGGCGTCTTCTTC	GGAGGCCATGAAAAGCCAAA
Fgf18	CCTGCACTTGCCTGTGTTTAC	TGCTTCCGACTCACATCATCT
Fgfr2	AATCTCCCAACCAGAAGCGTA	CTCCCCAATAAGCACTGTCCT
Fat4	TCAGCCAAACCGAGTCA	TCCATTACACAGCCCACA
Runx1	GATGGCACTCTGGTCACCG	GCCGCTCGGAAAAGGACAA
Dlk1	AGTGCGAAACCTGGGTGTC	GCCTCCTTGTTGAAAGTGGTCA
Runx2	GGCCGGGAATGATGAGAACTAC	GGACCGTCCACTGTCACTTT
Bmp4	TGATACCTGAGACCGGGAAG	AGCCGGTAAAGATCCCTCAT
Six1	GAAAGGGAGAACACCGAAAACA	GTGGCCCATATTGCTCTGGA
Gapdh	CATCACTGCCACCCAGAAGACTG	ATGCCAGTGAGCTTCCCGTTCAG

TABLE 3 List of number of input reads and uniquely mapped reads of RNA-sequencing.

Samples	Number of input reads	Uniquely mapped reads
C3H10_control_rep1	36750192	89.66%
C3H10_control_rep2	26716583	91.18%
C3H10_control_rep3	21658547	91.04%
C3H10_Six1kd_rep1	35031085	92.86%
C3H10_Six1kd_rep2	35952420	92.65%
C3H10_Six1kd_rep3	38199370	92.71%
Six1_WT_1204	38041711	87.9%
Six1_WT_1703	36188803	88.66%
Six1_KO_1201	40664817	88.61%
Six1_KO_1702	33502150	89.23%
